# Epitope-Evaluator: An interactive web application to study predicted T-cell epitopes

**DOI:** 10.1371/journal.pone.0273577

**Published:** 2022-08-26

**Authors:** Luis Fernando Soto, David Requena, Juan Ignacio Fuxman Bass

**Affiliations:** 1 Escuela Profesional de Genética y Biotecnología, Facultad de Ciencias Biológicas, Universidad Nacional Mayor de San Marcos, Lima, Perú; 2 Laboratory of Cellular Biophysics, The Rockefeller University, New York, NY, United States of America; 3 Biology Department, Boston University, Boston, Massachusetts, United States of America; The 8th Medical Center of PLA General Hospital, CHINA

## Abstract

Multiple immunoinformatic tools have been developed to predict T-cell epitopes from protein amino acid sequences for different major histocompatibility complex (MHC) alleles. These prediction tools output hundreds of potential peptide candidates which require further processing; however, these tools are either not graphical or not friendly for non-programming users. We present Epitope-Evaluator, a web tool developed in the Shiny/R framework to interactively analyze predicted T-cell epitopes. Epitope-Evaluator contains six tools providing the distribution of epitopes across a selected set of MHC alleles, the promiscuity and conservation of epitopes, and their density and location within antigens. Epitope-Evaluator requires as input the fasta file of protein sequences and the output prediction file coming out from any predictor. By choosing different cutoffs and parameters, users can produce several interactive plots and tables that can be downloaded as JPG and text files, respectively. Using Epitope-Evaluator, we found the HLA-B*40, HLA-B*27:05 and HLA-B*07:02 recognized fewer epitopes from the SARS-CoV-2 proteome than other MHC Class I alleles. We also identified shared epitopes between Delta, Omicron, and Wuhan Spike variants as well as variant-specific epitopes. In summary, Epitope-Evaluator removes the programming barrier and provides intuitive tools, allowing a straightforward interpretation and graphical representations that facilitate the selection of candidate epitopes for experimental evaluation. The web server Epitope-Evaluator is available at https://fuxmanlab.shinyapps.io/Epitope-Evaluator/

## Introduction

T-cell epitopes are peptides derived from processed antigens that are recognized by T-cells to elicit adaptive immune responses. Generally, CD4+ T-cells recognize epitopes between 13–17 amino acidic residues presented on the surface of major histocompatibility complex (MHC) class II molecules, while CD8+ T-cells recognize peptides of around 9 amino acid residues presented on the surface of MHC class I molecules [1]. This allows T-cells to detect pathogens and abnormal self-antigens from cancer cells. Epitopes can be used to detect the magnitude of epitope-specific T-cell responses in an input sample based on cytokine secretion assays such as ELISPOTs or ELISAs [2, 3]. Furthermore, the detection of epitope-specific T-cells has been used in diagnostic applications and to deimmunize proteins used as biological drugs [4, 5]. Additional interest in T-cell epitopes is related to the cancer immunotherapy field, where the number of potential T-cell neoepitopes in a tumor has been proposed as a marker of success for checkpoint blockade treatments, and where tumor-specific epitopes are being used to induce tumor-specific T-cell responses [6].

The Immune Epitope Database and Analysis (IEDB) resource is a comprehensive database of experimental epitopes derived from individual low-throughput and high-throughput studies [7]. There are three common categories of assays to identify T-cell epitopes considered by the IEDB. The first is assays measuring MHC binding in vitro to determine which peptides could be presented to T-cells [8]. The second assay is MHC ligand elution where ligands are identified by mass spectrometry [9]. The last assay consists in measuring T-cell response after the recognition of an epitope [10].

Given the complexity of experimental assays to measure epitope binding, several tools that predict epitope binding to different MHC alleles based on epitope amino acid sequences have been developed. Predictors such as SYFPEITHI [11] and BIMAS [12] use matrix-based methods making them fast algorithms but not the most accurate. More recent epitope predictors are based on machine learning algorithms trained with experimental data. These software such as NetMHC [13], NetMHCpan and NetMHCIIpan [14], and MHCFlurry [15] have shown to over-perform first generation methods. T-cell predictors provide a raw score (predicted binding score), and percentile rank (relative binding affinity) for each peptide, being percentile ranks the most widely used metric to filter T-cell epitopes [16].

**Fig 1:**
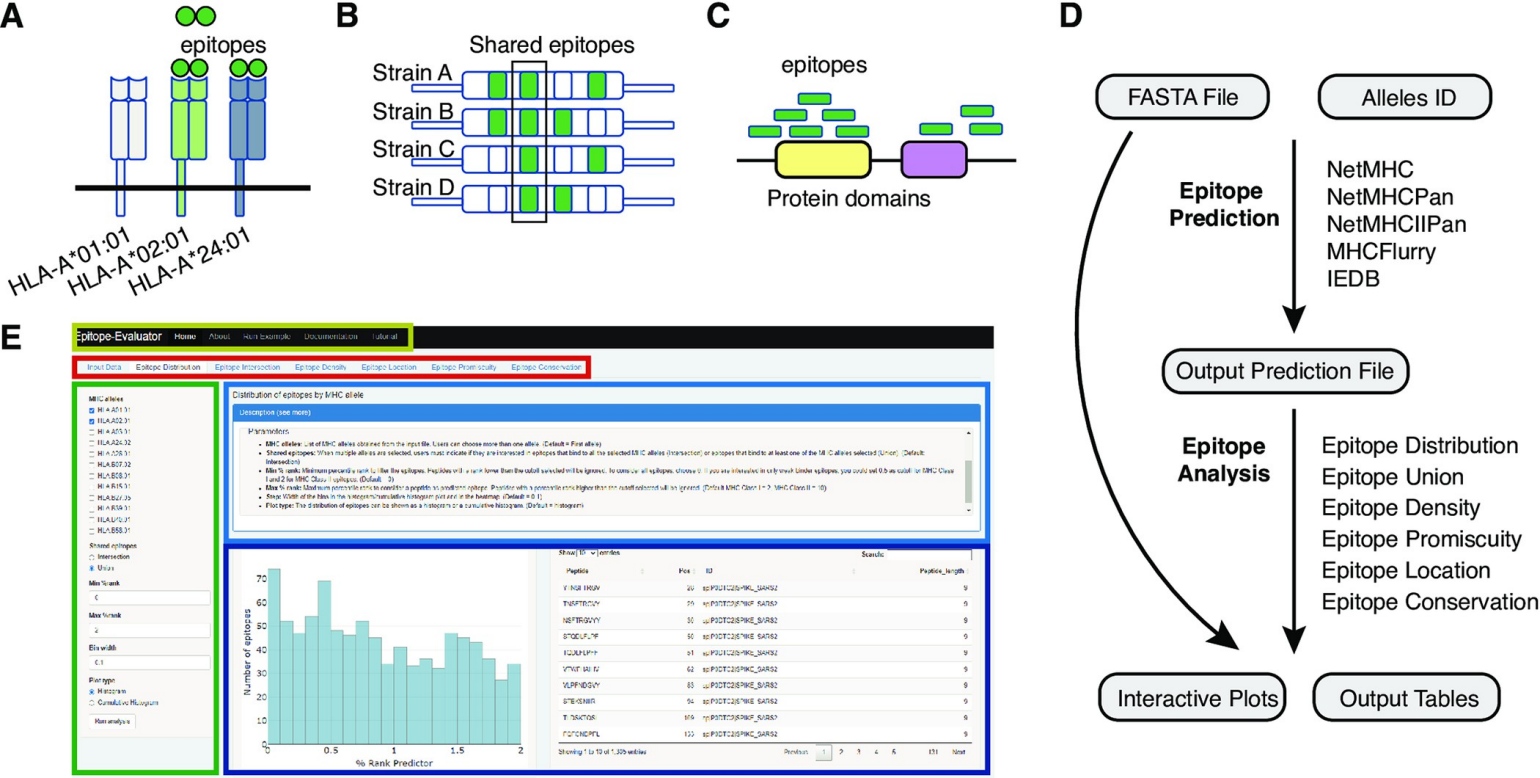
Schematic representation of characteristics of predicted epitopes and workflow of Epitope-Evaluator. **(A)** The promiscuity of epitopes represents their ability to be recognized by multiple Class I or Class II alleles. **(B)** The conservation of epitopes allows appropriate immune responses against several pathogen strains. Otherwise, restricted epitopes allow better discrimination across strains such as in immunological tests. **(C)** Determining the regions or domains enriched or depleted in epitopes helps to propose better peptide-based vaccines. Green circles and rectangles represent predicted epitopes. **(D)** Workflow to predict and analyze T-cell epitopes. All the current predictors of T-cell epitopes require a FASTA file containing protein sequences and the selection of MHC alleles of interest and retrieve an output prediction file containing a score for each epitope-allele pair (top). The Epitope-Evaluator requires the same FASTA file used as input in the prediction and the output file given by any predictor, and returns different plots and tables depending on the tool used (bottom). **(E)** Preview of the Epitope-Evaluator online web server. Rectangles indicate each section of the webserver. The title section (yellow), the tools section (red), the parameters section (green), the help section (blue), and the output section (purple).

These predictors often return a large number of predicted T-cell epitopes which need to be further filtered before experimental testing. Epitope features such as the predicted binding strength, the promiscuity (i.e., the number of MHC alleles they could bind to), the conservation across homologs, and the location within the amino acid sequence of the antigens (**Fig 1A–1C**) allow an adequate filtering and selection process. In particular, highly conserved epitopes may be good vaccine candidates as they would generate a cross-protective response, not only against the original pathogen but against other strains/types. For example, conserved epitopes against different influenza A and B subtypes have been targeted to elicit protective responses in mice [17]. A more recent study identified conserved epitopes in Spike which may be targeted to protect against emerging SARS-CoV-2 variants [18]. The identification of promiscuous epitopes can also help select vaccine candidates as promiscuous epitopes allow broader protection for populations with different MHC genotypes. These promiscuous epitopes have facilitated vaccine designs against different pathogens such as hepatitis C virus [19] and Plasmodium falciparum [20]. The analysis of the location of epitopes within antigens can also inform on the regions with high prevalence of epitopes which can be important for subunit vaccines. For example, a study found that the RBD region of Spike protein is enriched with conformational epitopes but lacked linear epitopes [21].

Although knowledge about epitope binding strength, promiscuity, conservation, and location are critical to understand adaptive immune responses and for rational vaccine designs, there are few software automatizing the selection and visualization process. EpitopeViewer [22] is a Java application for the visualization of immune epitopes in IEDB allowing the identification of epitopes within the 3D structure of antigens or immunological complexes; however, this application does not show information about promiscuity or conservation of epitopes, and it is not currently maintained. IEDB allows analyses such as population coverage, epitope conservancy, and other tools such as cluster analysis or mapping mimotopes to antigens [23]. However, most of these methods are not graphical and require some degree of programming experience, making them less accessible to non-programming researchers. Due to these shortcomings, we developed a user-friendly Shiny app named “Epitope-Evaluator”. This tool enables the analysis of T-cell epitopes such as the identification of conserved epitopes, promiscuous epitopes, or epitope-enriched protein regions focusing on the graphical interface; and facilitating its use for non-programming researchers.

### Design and implementation

Epitope-Evaluator is implemented in R v4.05 using the Shiny library. Input files are mainly processed with the dplyr package, and interactive plots are generated with ggplot2 and plotly packages. However, the app also requires the following additional libraries: readr, grid, gridExtra, reshape, shiny dashboard, tidyselect, rlist and tibble. Epitope-Evaluator is available online at https://fuxmanlab.shinyapps.io/Epitope-Evaluator/. The application can also be run locally by downloading the code on its GitHub repository https://github.com/SotoLF/Epitope-Evaluator and launched in Windows, Mac OS, and Linux distributions. In addition to the Web application, the code may be easily dissected and implemented within individualized pipelines by other research groups.

Epitope-Evaluator requires as input: 1) a multi-FASTA file containing the IDs and the sequences of the antigenic proteins, and 2) the respective prediction file previously obtained from a T-cell epitope predictor (**Fig 1D**). Currently, Epitope-Evaluator recognizes prediction files from NetMHC4.0 [24], NetMHCPan4.1 [14], MHCFlurry2.0 [25], IEDB-Consensus [7], and NetMHCIIPan2.3 [26]. However, users may indicate “other” if predictions are obtained from tools other than the ones listed. In this case, the prediction file should have the following columns: the peptide sequence, its position within the protein, the protein ID, the protein length, and subsequent columns corresponding to each of the MHC alleles evaluated, where each value indicates a score for each epitope (See Example section in the shiny app). In addition to this, users must indicate whether the score in the table corresponds to the “percentile rank” or “binding affinity score”. The applicative will automatically identify whether the epitopes are class I or class II based on the name of the MHC alleles.

The web server is composed of 6 different sections: ’Home’, ‘About’, ‘Run Example’, ‘Documentation’ and ‘Tutorial’. The ‘Home’ section comprises the Input tab and the 6 different tools available **(Fig 1E)**. Each of these tools is independent, thus, users can run all analyses in parallel. Each of the six tools has four different subsections: 1) The parameters section, 2) the title section, 3) the help section, and 4) the output section (**Fig 1E**). The parameter section, located on the left side of the web application, allows users to set different options and parameters for the corresponding tool. The help section describes the functionality of each tool, details each parameter, and explains the plots and tables returned in the output section. The output section shows the plots and tables from the selected analyses which are downloadable. All the tools contain interactive plots where users can zoom in/out, select regions, and obtain more information by hovering over the plots. The ‘About’ section shows a brief summary of Epitope-Evaluator and specifies the necessary files to run the shiny app. The ‘Run Example’ section contains an example data so users can try the different tools. The ‘Documentation’ section includes the link to the GitHub to download the code and information of the different T-cell epitopes predictors currently supported. In the ‘Tutorial’ section, we have included videos showing the different steps to use the shiny app and the interactivity of each tool.

### Description of the Epitope-Evaluator tools

Epitope-Evaluator facilitates the interpretation, visualization, and selection of predicted epitopes. This Shiny app can be used for analyzing MHC Class I and II epitopes and is compatible with any epitope predictor used. More information about the functionalities available in each section, main parameters, and main interface are available in the ‘Run Example’ section where it is also possible to download the sample dataset to use the shiny app. Epitope-Evaluator comprises the following six tools:

#### (1) Epitope distribution

**Fig 2:**
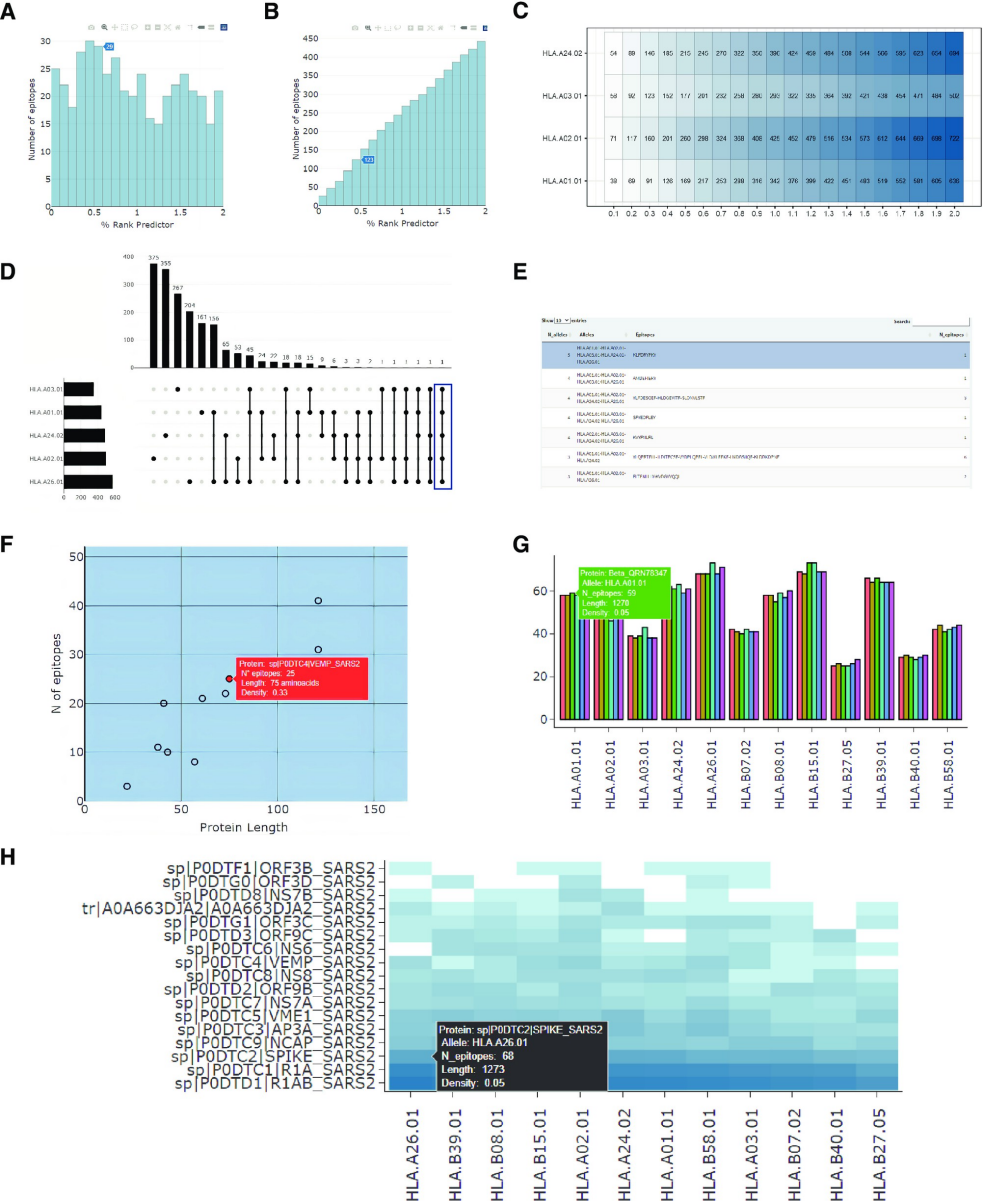
Outputs of the ‘Epitope Distribution’, ‘Epitope Intersection’, and ‘Epitope Density’ tools. Plots produced using the (**A-C**) ‘Epitope Distribution’, (**D, E**) ‘Epitope Intersection’, and (**F-H**) ‘Epitope Density’ tools. **(A)** Histogram showing the number of epitopes within a particular range of percentage rank. **(B)** The cumulative histogram shows the number of epitopes with a percentage rank lower than a specified value. Hovering over any of the columns shows the corresponding number of epitopes. **(C)** The number of epitopes predicted to bind to each MHC allele considering different cutoffs. The cell color intensity represents the number of epitopes. **(D)** Up-Set plot, produced by the ‘Epitope Intersection’ tool, showing the number of epitopes shared by different combinations of five MHC Class I alleles. The five selected alleles are on the left side and the number of epitopes in each region is at the top. Individual points in the grid indicate epitopes binding to a specific MHC allele, while connected points indicate epitopes that can bind to multiple MHC alleles. **(E)** The table shows, for each region in the Up-Set Plot, the epitope sequences, the MHC alleles to which they are predicted to bind, and the number of epitopes within each region. **(F)** Scatter plot between protein length and the number of MHC Class I epitopes from SARS-CoV-2 proteins. **(G)** Bar plot showing the number of epitopes of five proteins variants predicted to bind to each MHC allele. The color of each bar represents a different protein variant. **(H)** Heatmap showing the number of epitopes within the SARS-CoV-2 proteins per MHC Class I allele. The cell color intensity represents the number of epitopes. Hovering over any point, bar, or cell (in the heatmap) shows more information such as the protein name, the number of epitopes, the MHC allele ID, the length of the protein, and the epitope density.

This tool helps to identify which MHC Class I or Class II alleles recognize the least (and the greatest) number of epitopes and, therefore, which genotypes could be presenting a weaker T-cell response. For example, patients with MHC alleles recognizing fewer epitopes could be correlated with more severe symptoms during a viral infection. Conversely, patients with MHC alleles recognizing more neo-epitopes could be correlated with a better response during cancer progression or immunotherapies. The Epitope Distribution depicts a histogram representing the number of epitopes within a percentile rank or binding score range allowing an easy identification of the number of strong binder epitopes (lower rank percentile) and weak binder epitopes (higher rank percentile). This histogram can be shown per MHC allele or for the union or intersection of different MHC alleles, which can represent the number of epitopes that can be recognized by a heterozygote individual or the number of epitopes that are recognized by the alleles present in a population, respectively. Users can select between plotting a histogram (**Fig 2A**) or a cumulative histogram (**Fig 2B**). Hovering over any of the histogram bars provides the number of predicted epitopes with a percentile rank lower than a selected cutoff. In addition, the tool shows a heatmap indicating the number of epitopes predicted to bind to each allele with a percentage rank lower than a defined cutoff (**Fig 2C**).

#### (2) Epitope intersection

Different populations are represented by distinct combinations of MHC alleles. This tool enables users to identify the set of epitopes that could be used in epitope vaccines that are potentially recognized by most/all MHC alleles in the population, as well as to identify epitopes restricted to a particular set of MHC alleles. Users need to select the MCH alleles of interest and a %rank cutoff to identify epitopes. This tool shows the number of epitopes predicted to bind to different MHC allele combinations represented as a Venn Diagram or Up-Set plot if 6 or fewer MHC alleles are selected, or an Up-Set plot if more than 6 MHC alleles are selected (**Fig 2D**). In addition to the downloadable plots, the tool provides a table containing the epitope sequences and the number of epitopes within each combination of MHC alleles (**Fig 2E**).

#### (3) Epitope density

This tool can be used to determine the set of proteins containing a high number of predicted epitopes as a first step to finding potentially highly immunogenic proteins, which should then be confirmed experimentally. The tool displays a scatter plot of protein length versus the number of epitopes predicted to bind an MHC allele or combination of alleles (**Fig 2F**). The tool also shows the absolute number of epitopes within each protein predicted to bind to each MHC allele. This visualization can be displayed as a bar plot for a small number of proteins, (**Fig 2G)** or as a heatmap for several proteins (**Fig 2H)**. In both cases, users can modify the plots by changing the fill range (i.e., by number or by the density of epitopes) and by arranging the set of proteins.

#### (4) Epitope viewer

**Fig 3:**
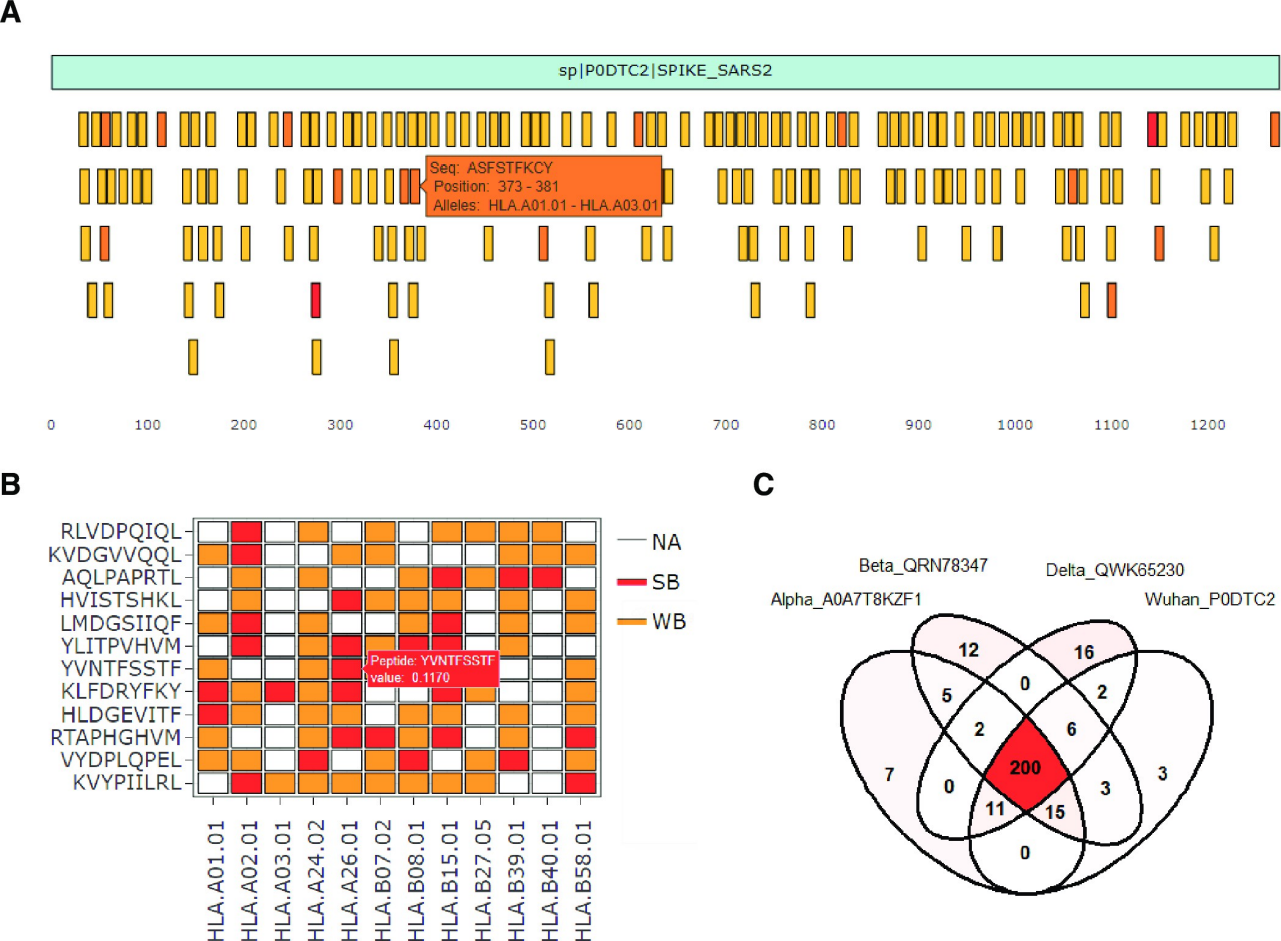
Plots produced with the *‘Epitope Viewer*, *‘Epitope Promiscuity’*, and *‘Epitope Conservation’* tools. **(A)** Linear representation of the SARS-CoV-2 Spike protein (light blue bar) and the MHC Class I epitopes (small bars). The color intensity of the represented epitopes varies from yellow to red indicating the number of MHC alleles predicted to bind to each epitope. Hovering over any epitope shows the sequence and the location of the epitope, and the MHC alleles to which the epitope is predicted to bind. **(B)** Heatmap showing the MHC Class I epitopes from the SARS-CoV-2 proteome that are predicted to bind at least seven MHC alleles. Red and yellow cells indicate strong (%rank < 0.5) and weak epitopes (%rank < 2), respectively. White cells indicate peptides that were not predicted as epitopes. **(C)** Venn diagram showing the shared epitopes found in the different Spike variants. The color intensity indicates the number of epitopes within each region.

This tool facilitates the visual identification of protein regions enriched or depleted with epitopes. The tool also helps to visualize how different coding mutations impact the increase or decrease of neo-epitopes within pathogenic proteins or oncogenic proteins. The tool can also be used to inform strategies to deimmunize peptide-based drugs by identifying potentially immunogenic epitopes which could then be mutated or deleted. Epitope Viewer graphically represents the position of each predicted epitope within each protein where more promiscuous epitopes are filled with a more intense color. Users must select a cutoff to identify epitopes and the MHC alleles of interest. Choosing the ‘Intersection’ option will show only epitopes predicted to bind to all of the selected MHC alleles. Selecting the ‘Union’ option will display all the epitopes which are colored ranging from yellow to red indicating the number of MHC alleles predicted to bind (**Fig 3A**).

#### (5) Epitope promiscuity

This tool facilitates the identification of promiscuous epitopes, which are epitopes predicted to bind to most MHC alleles, and their predicted percentage rank affinity regardless to which protein they belong. This tool allows to identify a list of epitopes that can cover all the MHC alleles of interest. Moreover, with proper selection of the parameters, users are able to perform analyses using only strong binding epitopes or all epitopes. Epitope Promiscuity shows in a heatmap the predicted epitopes that bind to more than a certain number of MHC alleles, set by the user. Users must also indicate the cutoff for both weak and strong binding epitopes. By default, these cutoffs are 0.5 and 2 for MHC Class I epitopes, and 2 and 10 for MHC Class II. If users prefer to analyze only strong binding epitopes, both cutoffs should be set with the same value (See Tutorial section). The output of this tool is a heatmap where strong binder and weak binder epitopes are indicated as red and orange, respectively. Moreover, the tool returns a table with the sequence of the promiscuous epitopes, their start position within the protein, and the name of the corresponding protein (**Fig 3B**).

#### (6) Epitope conservation

This tool allows users to identify epitopes that are present in multiple proteins, which can be useful to identify conserved epitopes across different pathogen strains. For example, this tool can be used to identify epitopes shared by all Spike variants from SARS-CoV-2. In addition, this tool allows for identifying epitopes gained or lost by diverse mutations. For this tool, users need to select the proteins, the MHC alleles of interest, and the cutoff percentile rank. The number of shared epitopes is represented as Venn Diagrams/Up-Set plot (≤ 6 proteins) or only Up-Set plot (> 6 proteins) (**Fig 3C**).

### Biological applications

To illustrate the use of Epitope-Evaluator, we analyzed the T-cell epitopes predicted from the SARS-CoV-2 proteome, identified the most antigenic SARS-CoV-2 proteins and regions, and evaluated the density of epitopes across the Spike protein from different SARS-CoV-2 variants. The MHC Class I epitopes were predicted using NetMHCPan [14] and MHCFlurry [25], and the MHC Class II epitopes, using NetMHCIIPan [14]. To identify strong binder and weak binder epitopes, we considered 0.5 and 2 as percentile rank cutoffs for MHC Class I epitopes, respectively. Similarly, we considered 2 and 10 as percentile rank cutoffs for MHC Class II epitopes. (**S1 File**).

### Analysis of predicted T-cell epitopes from the SARS-CoV-2 proteome

**Fig 4:**
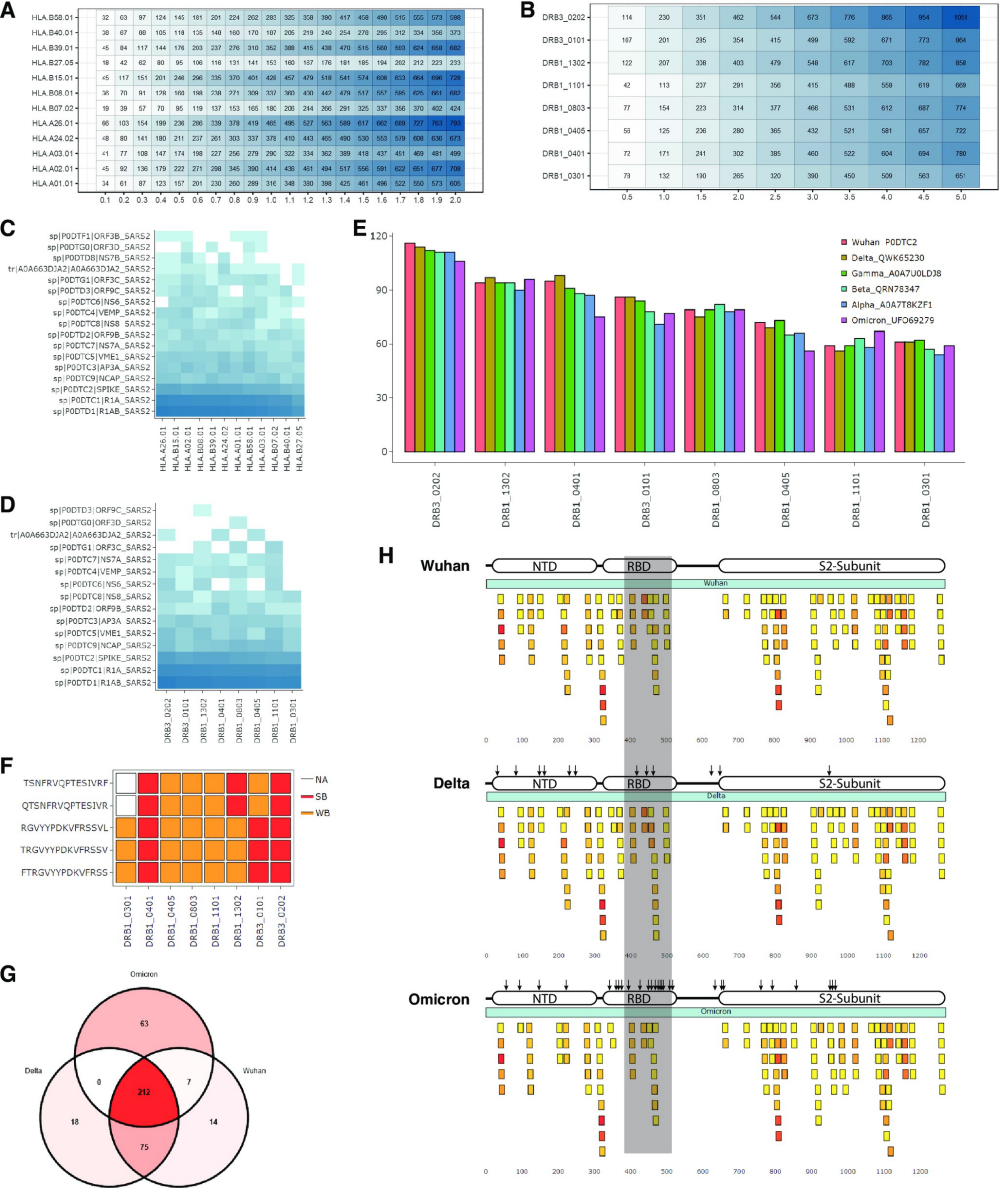
Analysis of MHC Class I and Class II epitopes from SARS-CoV-2 proteome and Spike variants. **(A, B)** Distributions of predicted epitopes across each **(A)** MHC class I and **(B)** class II alleles considering different percentage rank as cutoffs. **(C, D)** Heatmaps showing the number of epitopes in each protein predicted to bind to each **(C)** MHC class I or **(D)** MHC class II alleles. The color intensity represents a higher number of epitopes. **(E)** Bar plot showing the number of predicted epitopes within Wuhan Spike and its variants across eight MHC Class II alleles. **(F)** A heatmap representing the most promiscuous epitopes found in Spike protein. **(G)** Venn diagram showing the number of shared epitopes across Spike variants, and the number of gained and lost epitopes for Delta and Omicron variants. **(H)** Representation of Wuhan Spike, Delta Spike, and Omicron Spike, and their MHC Class II epitopes (%rank < 5). Spike proteins are shown as a light blue bar, while epitopes are represented as small bars colored from yellow to red representing the number of binding MHC alleles. A shadowed area is indicating the region where most. differences are located (amino acids 400–500).

The prediction of MHC Class I epitopes from the SARS-CoV-2 proteome resulted in 1,150 strong binder and 1,645 weak binder epitopes, predicted to bind to at least one MHC allele. However, using the “*Epitope Distribution*” tool, we found that each MHC allele recognized a different number of epitopes (**Fig 4A**). Although most MHC Class I alleles recognized around 200 strong binder and 400 weak binder epitopes, the HLA-B*27:05, HLA-B*40:01, and HLA-B*07:02 showed the lowest number of predicted epitopes (~100 strong binder and ~200 weak binder epitopes). Moreover, this observation was not dependent on the percentile rank selected as the same low binder MHC alleles were identified across different percentile rank cutoffs **(Fig 4A)**. This suggests that patients with these alleles may have a weaker cytotoxic response against viral infection. The HLA-B*40 allele has been correlated with moderate/severe disease course [27]; however, less is known about the HLA-B*27:05 and HLA-B*07:02.

We also predicted epitopes binding to MHC Class II, which resulted in 2,245 weak binder and 1,091 strong binder epitopes. Contrary to what we observed for MHC Class I alleles, a similar number of epitopes was predicted to bind each MHC Class II allele regardless of the cutoff selected (**Fig 4B**). This suggests that variability in MHC Class II alleles is less likely to be correlated with a worse or better viral response against SARS-CoV-2.

### Identification of potentially antigenic SARS-CoV-2 proteins

As a first step to design recombinant vaccines, we identified the SARS-CoV-2 proteins enriched in MHC Class I and Class II epitopes, using the ‘Epitope Density’ tool. As expected, we found that R1AB and R1A, which are the largest SARS-CoV-2 proteins, contained the highest number of MHC Class I epitopes in total (2,028 and 1,261 epitopes, respectively). From the remaining proteins, we show that Spike (S), Nucleocapsid (N), ORF3A and Membrane (M) proteins contain the highest number of MHC-Class I. Moreover, these proteins contain at least one MHC Class I epitope predicted to bind to each MHC allele, which suggests a broader protection (**Fig 4C**).

Similarly, R1AB and R1A contained the highest number of MHC Class II epitopes in total (1,546 and 910 epitopes, respectively). From the remaining, we found that S, N, M and ORF3A also contained the highest number of MHC-Class II epitopes. These proteins also contained at least one epitope predicted to bind to each MHC-Class II allele (**Fig 4D**). Altogether, these results suggest that proteins N, M and ORF3 could also be considered complementary vaccine antigens, as previously suggested [19–23].

### Analysis of epitopes within variants of the Spike protein

We predicted MHC Class II epitopes from the Alpha, Beta, Gamma, Delta, Omicron and Wuhan Spike variants (**S1 File**) and determined whether MHC Class II alleles were predicted to recognize fewer epitopes due to the mutations in each variant by using the “Epitope Density” tool. We found that Wuhan Spike contains a similar number of epitopes as other Spike variants for each MHC Class II allele (**Fig 4E**), suggesting that mutations in current variants do not have a marked impact on the recognition of epitopes by MHC class II alleles. We then used the ‘Epitope Promiscuity’ tool to identify highly promiscuous epitopes and determine whether these epitopes were affected by the mutations in the Spike variants. We identified five epitopes binding to at least 7 MHC Class II alleles and all five were present in the six Spike variants, suggesting that these epitopes may be well suited for epitope vaccines as they are recognized by most MHC Class II alleles and are present in all current variants (**Fig 4F**).

Next, we evaluated whether MHC Class II epitopes identified from Wuhan Spike are conserved in the variants of concern (Delta and Omicron variants). These epitopes could suggest cross-protection across variants when immunized against Wuhan Spike as it is the antigen used in most current SARS-CoV-2 vaccines. Using the ‘Epitope Conservation’ tool we determined the number of epitopes shared, gained, and lost, by mutations in Delta and Omicron. We identified 212 epitopes shared across the three variants, 21 and 18 epitopes were lost and gained in the Delta variant, and 89 and 63 epitopes were lost and gained in the Omicron variant, respectively (**Fig 4G**). This is consistent with the higher number of mutations in Omicron Spike compared to Delta Spike [28, 29]. We then used the ‘Epitope Viewer’ tool to determine the amino acid region within Spike associated with gained and lost epitopes due to mutations. We found that in the three variants, the region between amino acids 508 and 658 is depleted of epitopes. We also found that most differences between variants are located in the region between amino acids 400 and 500 corresponding to the Receptor-binding motifs in the S1 region which is frequently mutated in Omicron (**Fig 4H**). Altogether, our analyses suggest that, although Spike variants can escape from certain antibodies elicited by vaccines against the Wuhan and Omicron Spike, the T-helper response may remain efficient, as demonstrated in other works [30, 31].

### Discussion and future directions

The identification of T-cell epitopes is an essential step to understand immune responses and for rational vaccine design. Although current software can produce hundreds of T-cell epitope predictions, these tools are generally not user-friendly. In this study, we present Epitope-Evaluator, a useful open-source program that allows the analysis and selection of candidate T-cell epitopes. Epitope-Evaluator is optimized to perform analyses of epitopes predicted from whole proteome as shown for SARS-CoV-2. Epitope-Evaluator supports most currently used predictors [16] to streamline prediction and analysis of T-cell epitopes. Further, its versatility allows Epitope-Evaluator to analyze outputs from any predictor, provided outputs are converted to the specified format, which extends the applicability of Epitope-Evaluator as new epitope prediction tools are developed.

The Epitope-Evaluator is comprised of six tools to perform a comprehensive epitope analysis showing interactive plots and downloadable results. These tools can be applied to different biological applications, including the identification of proteins and regions to design peptide-based vaccines, the identification of promiscuous and conserved epitopes for the development of multi-epitope vaccines, and the study of the impact of mutations in the creation of neo-epitopes among others. It is important to note that, although Epitope-Evaluator considers epitope binding strength, location, and other sequence-based parameters, other aspects associated with the biological function of each protein need to be considered for rational vaccine design. This includes location of the proteins within the pathogen structure (e.g., surface versus internal), expression level, and potential toxicity. These parameters are case-specific and are therefore not considered by Epitope-Evaluator.

As an example, we used Epitope-Evaluator to analyze the MHC Class I and Class II epitopes from the SARS-CoV-2 proteome. Our analysis confirmed previously reported results such as the potential of N and M proteins as complementary candidate vaccines [32, 33]. Our analyzes also identified new MHC Class I alleles that recognized fewer epitopes. Further studies stratifying patients based on MHC alleles are needed to determine whether these alleles are associated with a reduced T-cell response and severe infection. We also determined the conserved epitopes among the different Spike variants and identified shared and variant-specific epitopes. Overall, mutations in Spike do not seem to have a marked impact on T-cell epitopes but rather on B-cell epitopes as has been previously shown [34, 35].

Future directions include adding new T-cell predictors, developing tools for analyzing B-cell epitopes and new ways to integrate MHC Class I T-cell epitopes, MHC Class II T-cell epitopes, and B-cell epitopes. Moreover, gene expression data for the antigens can be included to prioritize epitopes of highly expressed genes. Altogether, this tool will assist immunologists and experimental scientists to interpret and analyze T-cell epitopes.

## Supporting information

S1 File Methods used to predict MHC Class I and II epitopes in different SAARS-CoV-2 strains and epitope analyses using Epitope-Evaluator. (DOCX)
