# Supplementary material for: Epitope-Evaluator: An interactive web application to study predicted T-cell epitopes
Source: PLoS One. 2022 Aug 26;17(8):e0273577. doi: 10.1371/journal.pone.0273577 (PMC9417011; doi:10.1371/journal.pone.0273577)
Supplement: S1 File — (DOCX) [file pone.0273577.s001.docx]

**Supplementary Document 1**

**Predictions of MHC Class I  and Class II epitopes**

We downloaded the proteome of SARS-CoV-2 from UNIPROT (ID: UP000464024) and used NetMHCPan 4.1 [1] and MHCflurry 2.0 [2] to predict 9-mer MHC Class I epitopes against supertype alleles: HLA-A*01:01, HLA-A*02:01, HLA-A*03:01, HLA-A*24:02, HLA-A*26:01, HLA-B*07:02, HLA-B*08:01, HLA-B*27:05, HLA-B*39:01, HLA-B*40:01, HLA-B*58:01, HLA-B*15:01. The prediction in NetMHCPan was performed using the online server, while for MHCflurry we used the following command line: mhcflurry-predict-scan proteins.fasta --alleles HLA-A*01:01 HLA-A*02:01 HLA-A*03:01 HLA-A*24:02 HLA-A*26:01 HLA-B*07:02 HLA-B*08:01 HLA-B*27:05 HLA-B*39:01 HLA-B*40:01 HLA-B*58:01 HLA-B*15:01 --peptide-lengths 9 --no-throw --results-all --out Results_MHCFlurry.txt. To be conservative, we considered as MHC Class I predicted epitopes those peptides that showed a %rank less or equal than 2 in both predictors for the same MHC allele and assigned the highest %rank as its global %rank. A customized R script was used to filter both files and merge into one file to be used as input for Epitope-Evaluator. Similarly, we predicted 15-mer MHC Class II epitopes using NetMHCIIPanII 4.0 [1] considering a % rank cutoff of 5, and the following alleles DRB1-03:01, DRB1-04:01, DRB1-04:05, DRB1-08:03, DRB1-11:01, DRB1-13:02 and DRB3-01:01, and DRB3-02:02.

**Analysis of  MHC Class I and II epitopes from the whole proteome of SARS-CoV-2**

To run the Epitope-Evaluator with MHC-Class I predicted epitopes, we uploaded the input file and selected the option ‘Others’ as the predictor, since our file comes from a combination of two predictors, and the %rank as Score Type. Then we clicked on ‘Run Analysis’. We used the *Epitope Distribution* tool and used the following parameters. All alleles were selected, ‘Union’ was considered in Shared epitopes. The min %rank, max %rank, and the step were 0, 2, and 0.1, respectively. The Plot Type was ‘Cumulative Histogram’. We used the *Epitope Density* and selected 2 as Cutoff %Rank, Heatmap as Plot Type, Descendent as Sort Type, and Epitopes Number as Color by options. Then, we clicked on ‘Run analysis’ and ‘Generate plot’.

For MHC-Class II predicted epitopes, we uploaded the input file and the fasta file. We selected the ‘NetMHCIIpan’ as the predictor and ‘Rank’ as the Score Type.  Then we clicked on ‘Run Analysis’. We used the *Epitope Distribution* tool and used the following parameters. All alleles were selected, ‘Union’ was considered in Shared epitopes. The min %rank, max %rank, and the step were 0, 5, and 0.5, respectively. The Plot Type was ‘Cumulative Histogram’. We used the *Epitope Density* and selected 5 as Cutoff %Rank, Heatmap as Plot Type, Descendent as Sort Type, and Epitopes Number as Color by options. Then, we clicked on ‘Run analysis’ and ‘Generate plot’.

**Prediction of  MHC Class II epitopes from Spike variants**

We downloaded the amino acid sequences of Spike variants from the NCBI using the following Genbank accession IDs (Alpha = QWE88920, Beta = QRN78347,  Gamma = QVE55289, Delta = QWK65230, Omicron = UFO69279.1) and predicted the MHC Class II epitopes as in the previous analysis.  We used NetMHCIIPanII 4.0 considering a % rank cutoff of 5, and the following alleles DRB1-03:01, DRB1-04:01, DRB1-04:05, DRB1-08:03, DRB1-11:01, DRB1-13:02 and DRB3-01:01, and DRB3-02:02.

**Analysis of  MHC Class II epitopes from Spike variants**

To run the Epitope-Evaluator, we uploaded the input file and the respective fasta file. We selected the ‘NetMHCIIpan’ as the predictor and ‘Rank’ as the Score Type.  Then we clicked on ‘Run Analysis’. We used the *Epitope Density* and selected 5 as Cutoff %Rank, Bar plot as Plot Type, Descendent as Sort Type, and Epitopes Number as Color by options. Then, we clicked on ‘Run analysis’ and ‘Generate plot’.  Then, we used the *Epitope Promiscuity* tool and selected 7 as the Minimum number of alleles, 1 and 5 as the Strong Binding Cutoff %rank, and the Weak Binding Cutoff %rank, respectively. Then, we clicked on ‘Run analysis’. To make the Venn Diagram, we used the *Epitope Conservation* tool. We selected ‘Wuhan, Delta, and Omicron’ as Proteins, 5 as Cutoff,  all the alleles, and Venn Diagram as the Plot Type. Then, we clicked on ‘Run analysis’. Lastly, we used the *Epitope Location* tool. We selected the variant of interest (Wuhan, Delta, and Omicron), 5 as Cutoff %Rank, all the alleles, and union as ‘Shared epitopes. Then, we clicked on ‘Run analysis’

**References**

1. Reynisson B, Alvarez B, Paul S, Peters B, Nielsen M. NetMHCpan-4.1 and NetMHCIIpan-4.0: improved predictions of MHC antigen presentation by concurrent motif deconvolution and integration of MS MHC eluted ligand data. Nucleic Acids Res. 2020;48(W1):W449-W54.

2. O'Donnell TJ, Rubinsteyn A, Laserson U. MHCflurry 2.0: Improved Pan-Allele Prediction of MHC Class I-Presented Peptides by Incorporating Antigen Processing. Cell Syst. 2020;11(1):42-8 e7.
